# Comparative analysis of the repertoire of insulin-reactive B cells in type 1 diabetes-prone and resistant mice

**DOI:** 10.3389/fimmu.2022.961209

**Published:** 2022-10-04

**Authors:** Maureen Banach, Isaac T. W. Harley, Andrew Getahun, John C. Cambier

**Affiliations:** ^1^ Department of Immunology and Microbiology, School of Medicine, University of Colorado Anschutz Medical Campus, Aurora, CO, United States; ^2^ Division of Rheumatology, Department of Medicine, School of Medicine, University of Colorado Anschutz Medical Campus, Aurora, CO, United States; ^3^ Rheumatology Section, Medicine Service, Rocky Mountain Regional Veterans Affairs Medical Center, Aurora, CO, United States

**Keywords:** B cell receptor (BCR), immunoglobulin light chain, BCR affinity, non-obese diabetes (NOD) mice, insulin, type 1 diabetes (T1D)

## Abstract

Seropositivity for autoantibodies against multiple islet antigens is associated with development of autoimmune type 1 diabetes (T1D), suggesting a role for B cells in disease. The importance of B cells in T1D is indicated by the effectiveness of B cell-therapies in mouse models and patients. B cells contribute to T1D by presenting islet antigens, including insulin, to diabetogenic T cells that kill pancreatic beta cells. The role of B cell receptor (BCR) affinity in T1D development is unclear. Here, we employed single cell RNA sequencing to define the relationship between BCR affinity for insulin and B cell phenotype during disease development. We utilized immunoglobulin (Ig) heavy chain (VH125) mouse models in which high-affinity insulin-reactive B cells (IBCs) were previously shown to be anergic in diabetes-resistant VH125.C57BL/6-H2g7 and activated in VH125. NOD mice developing disease. Here, high-affinity IBCs were found in the spleen of prediabetic VH125. NOD mice and exhibited marginal zone or follicular phenotypes. Ig light chains expressed by these B cells are unmutated and biased toward Vκ4-74 and Vκ4-57 usage. Receptors expressed by anergic high-affinity IBCs of diabetes-resistant VH125.C57BL/6-H2g7 are also unmutated; however, in this genetic background light chains are polymorphic relative to those of NOD. Light chains derived from NOD and C57BL/6-H2g7 genetic backgrounds conferred divergent kinetics of binding to insulin when paired with the VH125 heavy chain. These findings suggest that relaxation of tolerance mechanisms in the NOD mouse leads to accumulation and partial activation of B cells expressing germline encoded high-affinity BCRs that support development of autoimmunity.

## Introduction

Type 1 diabetes (T1D) involves T cell-mediated destruction of pancreatic beta cells leading to life-threatening insulin deficiency. Over the past two decades, B cells have been increasingly recognized as important contributors to T1D. The roles of B cells in T1D appear to involve presentation of islet antigens to diabetogenic T cells, possibly secretion of cytokines and production of pancreatic islet antibodies (1–3). A harbinger of T1D development is seropositivity for a combination of antibodies reactive with pancreatic islet antigens such as insulin, insulinoma-associated antigen-2, and glutamic acid decarboxylase 65 (4). Indeed, the majority of at-risk children who develop seropositivity to multiple islet autoantibodies progress to diabetes (5, 6). However, autoantibody production appears to be an epiphenomenon relative to disease development. While deficiency of B cells prevents diabetes, B cells that are unable to secrete antibodies support disease development in the non-obese diabetic (NOD) model (7, 8). Furthermore, therapeutic depletion of B cells with anti-CD20 antibody is effective in delaying disease progression despite the fact that it does not target the main antibody producers – plasma cells (9, 10).

Binding of B cell antigen receptors (BCR) to a cognate antigen initiates a signaling cascade leading to B cell activation, antigen presentation to T cells, cytokine production, and secretion of antibodies. The secreted antibodies typically have specificity identical to that of BCR of the antibody-secreting B cell’s precursor. BCR immunoglobulins (Ig) are composed of heavy and light chains comprising distinct regions: constant regions and more diverse variable regions that determine antigen reactivity. During early B cell development, the BCR variable region is assembled by somatic recombination of gene segments: variable (V), diversity (D), and joint (J) in the heavy chain and V and J in the light chain. The combinatorial nature of VDJ recombination and addition and/or removal of nucleotides between the segments generates a repertoire of different heavy and light sequences that confer reactivity to antigens. BCR binding strength to antigen can be assessed by affinity and avidity. While affinity estimates the binding strength at a single binding site to its antigen, avidity measures the sum of all interactions. Theoretically, BCR with higher affinity binds antigen at lower concentrations. In addition, BCR binding strength depends on antigen valence as the BCR interaction with a monomeric antigen is defined by affinity but with a polymeric antigen by avidity.

While central tolerance is induced in the bone marrow, peripheral tolerance is induced in distal sites. In the bone marrow, immature B cells that bind autoantigen with high avidity are triggered to modify their BCR specificity by receptor editing and if failing this they are eliminated by clonal deletion. Low avidity interactions of immature B cells with autoantigen does not lead to this central tolerance mechanism. Instead, these B cells proceed to the periphery where they are restrained by either ignorance, wherein they are unaware of antigen in their environment due to its low concentration or by anergy. In contrast to ignorance, anergy is an active process wherein chronic low avidity antigen interaction with B cells induces unresponsiveness to further stimulation. In T1D, the mechanisms of central and/or peripheral tolerance appear defective, resulting in increased numbers of autoreactive B cells in the periphery and production of autoantibodies (11, 12). Specifically, it has been reported that anergy is lost in insulin-reactive B cells (IBCs), both in diabetes-prone NOD mice and people developing diabetes (13, 14).

B cell reactivity to islet antigens is critical for diabetes development. NOD mice in which BCR reactivity is biased toward insulin or neural antigen peripherin develop diabetes at an earlier age than non-transgenic counterparts (15, 16). While NOD mice with a diverse repertoire develop diabetes spontaneously on average by 18 weeks of age, NOD mice that do not express islet-antigen-reactive BCR by virtue of immunoglobulin transgenesis do not develop disease (15, 17). In the case of T1D driven by IBCs, BCR affinity is likely a critical factor in determining risk as insulin circulating in blood is monomeric and at low concentration (10 × 10^-10^ M) (18). Such conditions should not support induction of B cell anergy or activation because of the low avidity of B cell-insulin interactions. Instead, these responses may be limited to B cells that have very high affinity to insulin and encounter polymeric insulin released by damaged islet beta cells (19). Alternatively, avidity requirements may be met if BCR recognizes insulin associated with ubiquitous insulin receptors expressed on the surface of most cells; however, it is noteworthy that the diabetogenic anti-insulin hybridoma mAb125 was reported to not recognize receptor-associated insulin (20).

NOD mice expressing transgenes encoding both heavy and light chains of the insulin-reactive 125 hybridoma (125Tg – the same as mAb125, 125Tg.NOD), only its VH125 heavy chain (VH125.NOD), or NOD mice with VH125 directed to the native IgH locus (VH125.SD.NOD) contain detectable levels of IBCs that can be subjected to phenotypic and affinity characterization. Although IBCs derived from 125Tg.NOD bind insulin with a high affinity of 3 × 10^-8^ M, these mice develop diabetes similarly to non-transgenic NOD counterparts (20, 21). Surprisingly, VH125.NOD and VH125.SD.NOD develop diabetes at an accelerated rate and with higher penetrance than in 125Tg or non-transgenic NOD (15, 21–23). This may indicate that disease development is facilitated when multiple autoreactive BCR clones participate. BCR of IBCs in VH125.NOD mice have a range of affinity for insulin from low 3.8 × 10^-6^ M in EW6 mAb to high 6.6 × 10^-9^ M in A12 mAb (24). Since the same VH125 heavy chain transgene is used by all of the IBCs in this model, it seems likely that they all recognize the same insulin epitope (15, 20, 25). How a semi-polyclonal BCR repertoire with a range of affinities for the same epitope enhances disease progression in VH125.NOD remains enigmatic.

Spleens of both VH125.NOD and VH125.C57BL/6-H2g7 harbor two distinct IBC populations distinguished by relative insulin binding and membrane-bound IgM (mIgM) expression (23). High-binding (high-affinity) IBCs of VH125.NOD express elevated levels of IgM compared to their counterparts in diabetes-resistant VH125.C57BL/6-H2g7 (23). In addition, high-affinity IBCs in VH125.NOD, but not in VH125.C57BL/6-H2g7, increase in number with age and disease progression (23). These features are consistent with an activated IBC phenotype in the former and anergic IBC phenotype in the latter (14). We have also reported that light chain retrogenic VH125 B cells expressing a high-affinity but not a low-affinity insulin-binding BCR relocate to pancreatic lymph nodes upon an adoptive transfer, suggesting that BCR affinity for insulin may influence B cell trafficking to the site of disease (24). In healthy humans, anergic IBCs express diverse BCR, and it is likely that the BCR repertoire and affinities of their activated counterparts in individuals with new-onset diabetes is restricted (13). Early characterization of the NOD IBC repertoire suggested a breadth of IBC affinity; however, it is unclear whether the repertoire of the activated and anergic high-affinity IBCs are dominated by variable regions with a unique range of affinities (26). Without knowledge of *in vivo* frequencies of IBCs expressing a particular BCR clonotype, transcriptomic profile of these cells, and functional affinity assays, understanding the role of BCR affinity in diabetes remains limited.

Here, we used single-cell RNA sequencing (scRNA-seq) to profile the BCR repertoire and phenotype of high-affinity IBCs derived from prediabetic VH125.NOD. The methodology involves encapsulation of single B cells in emulsion droplets along with nucleotide barcodes and reagents necessary for cDNA synthesis, and single cell library construction. The scRNA-seq method enables simultaneous recovery of paired BCR light and heavy chain sequencing and cellular transcriptome. High-affinity IBCs with marginal zone (MZ) and follicular (FO) gene signature were found in the spleen of prediabetic VH125.NOD. The light chain repertoire of high-affinity IBCs was overrepresented by germline Vκ4-74 and Vκ4-57 in prediabetic VH125.NOD. Additionally, we profiled anergic high-affinity IBCs from diabetes-resistant VH125.C57BL/6-H2g7 using traditional PCR approaches. These cells were unmutated but polymorphic relative to NOD counterparts. We generated 45 recombinant antibodies (recAbs) containing VH125 and light chains derived from either NOD or C57BL/6-H2g7 genetic backgrounds for the analysis of insulin affinity. RecAbs containing Vκ4-74 light chain bound insulin with higher affinity compared to BCR comprising other light chains. Together, this report highlights the role of BCR affinity on functional status of pathogenic B cells in autoimmune settings.

## Methods

### Mice

Mice expressing IgM^a^ VH125 heavy chain transgene [Cg-Tg(Igh-6/Igh-V125)2Jwt/JwtJ] on NOD/ShiLtJ were generously provided by J.W. Thomas (Vanderbilt University, TN, USA). VH125.C57BL/6 mice were crossed with C57BL/6-H2g7 [B6.NOD-(D17Mit21-D17Mit10)/LtJ] (Jackson Labs, Bar Harbor, ME, USA) to generate VH125.C57BL/6-H2g7 mice expressing NOD-derived MHC class II allotype which facilitates insulin presentation to autoreactive T cells. All experiments involved 8- to 12-week-old female VH125.NOD mice indicated to be prediabetic by two consecutive blood glucose readings within a range of 150-200 mg/dl (OneTouch Ultramini meter, OneTouch, Wayne, PA, USA) and age-matched female VH125.C57BL/6-H2g7. All animal experiments were performed in accordance with the regulations of the Institutional Animal Care and Use Committees at the University of Colorado Anschutz Medical Campus.

### Tissue preparation

Single cell suspensions from spleen were prepared using cell strainers. Ammonium chloride-potassium buffer was used to lyse erythrocytes. Dead cells were removed with Dead Cell Removal Kit (Miltenyi Biotec). Cells were filtered with cell strainers, spun down, and resuspended in FACS (PBS/0.5% BSA) buffer.

### Enrichment of insulin-reactive B cells (IBCs) for flow cytometry and fluorescence-activated cell sorting (FACS)

Splenocytes were treated with 2.4G2 anti-Fc γRIIB antibody (Cambier lab) to block FcR binding. In primary staining, cells were incubated with anti-B220 (BD, RA3-6B2) and anti-IgM antibodies (Cambier lab, b-7-6), and 0.1μg biotinylated human recombinant insulin (Sigma) for 30min on ice. In secondary staining, cells were stained with 2µg Alexa Fluor™ 647 conjugated streptavidin (Invitrogen) for 20min on ice. Cells were washed and resuspended in MACS buffer (PBS/0.5% BSA/2 mmol/L EDTA), and incubated with anti-Cy5/anti-Alexa 647 microbeads (Miltenyi Biotec) for 15min at 4°C. To enrich IBCs, cells were passed through LS column mounted on a magnet (Miltenyi Biotec). To distinguish kappa (Igκ) and lambda (Igλ) light chains, cell markers such as: anti-Igκ (SouthernBiotech) and anti-Igλ (SouthernBiotech) were used during primary staining. Flow cytometry and cell sorting were performed using an LSR Fortessa X-20 (BD) and FACS on MoFlo Astrios EQ (Beckman Coulter). Data were analyzed with FlowJo software version 9 (Ashland, OR, USA).

### Cellular barcoding

During primary staining, splenocytes were additionally labeled with TotalSeq-C antibodies (BioLegend): prediabetic VH125.NOD mouse 1 – TotalSeq-CO301 hashtag 1 with 5′– ACCCACCAGTAAGAC–3′ barcode, mouse 2 – TotalSeq-CO302 hashtag 2 with 5′– GGTCGAGAGCATTCA–3′ barcode, and mouse 3 – TotalSeq-CO303 hashtag 3 with 5′– CTTGCCGCATGTCAT–3′ barcode. Labelling with hashtag antibodies was performed on ice for 30 min. After sorting, high-affinity IBCs from three VH125.NOD mice were pooled for library construction using 10X Genomics protocols and Illumina sequencing.

### Immunoglobulin light chain (IgL) identification by single cell RNA sequencing

IBCs were obtained as described above. Additionally, splenocytes from different mice were barcoded with unique hashtag-antibodies during primary staining (BioLegend, see *Methods: Cellular barcoding* above). High-affinity IBCs were sorted into PBS supplemented with 10% FBS, counted, and assessed for cell viability. Cells were diluted into a final concentration of 400 cells/µl in 50μl volume. Single-cell RNA-sequencing (scRNA-seq) was performed with 10X Genomics technology and protocols. Briefly, about 7,000 cells were loaded onto the Chromium Controller microfluidics device and cells were encapsulated in emulsion-droplets containing beads and cDNA synthesis reagents (10X Genomics). Three sequencing libraries were prepared: 5′ total gene expression, hashtag, and VDJ antibody, according to the manufacturer’s instructions. Briefly, reverse transcription (RT) of total mRNA occurred in each individual droplet to generate cDNA. Simultaneously, hashtag barcodes were captured and overlap-extended with mRNA transcripts within the same droplet to generate DNA. After RT, droplets were broken, and the cDNA and DNA were amplified by PCR, purified, and separated with size selection beads. VDJ was amplified with additional PCR with mouse B cell Chromium Single-Cell V(D)J Enrichment Kit (10x Genomics). Sequencing was performed on a NovaSEQ 6000 system (Illumina). Raw base call files were processed with the 10x Genomics Cell Ranger pipeline package (version 3.1.0) using a standard protocol and with mouse reference genomes: mm10 (GENCOD vM23/Ensemble 98) and vdj-GRCm38-alts-ensembl-3.1.0 available on the 10xGenomics website. The Cell Ranger package demultiplexed raw base call files into FASTQ files, aligned, filtered cells using quality metrics, counted nucleotide barcodes, performed gene expression analysis in reference to VDJ sequences and hashtag data. The total number of reads assigned to the gene expression library was 288,478,010 and VDJ – 200,957,192. Fraction of reads with valid barcodes and unique molecular identifiers was 91.5% for gene expression and 96.7% for VDJ library. Sequencing saturation reflecting library complexity as well as sequencing depth reached 88.8%. Mean read pairs per cell were 45,287 for gene expression and hashtag library and 36,873 for antibody VDJ library. Further analysis was performed with 10x Genomics software: Loupe Cell and Loupe VDJ Browsers.

### Transcriptional signatures of IBCs

Three transcriptional subclusters among high-affinity IBCs were determined by unsupervised clustering method *via* 10x Genomics Cell Ranger pipeline with mouse reference transcriptome and visualized by 10x Loupe Browser software (10X Genomics). Fifty-two genes significantly differentiated cluster 1, 2, and 3 with a p-value of <0.05. Custom Python 3.9.7 script (https://github.com/harleyi/InsulinBindingBCells2022) was applied to visualize transcriptomic signatures between three clusters. Next, based on gene name, these fifty-two differentiating genes were merged to genes in the Immunological Genome Project (ImmGen) Ultra-Low-Input (ULI) Systemwide RNA-seq profile (#1) and normalized to gene count table (https://sharehost.hms.harvard.edu/immgen/GSE122597/GSE122597_Normalized_Gene_count_table.csv) (27). Fifty out of fifty-two genes were successfully mapped to the ImmGen cell atlas. For each gene, the median gene expression within a given population was then normalized according to average and standard deviation of that gene for all splenic and germinal center B cell populations. Significantly up- and down-regulated genes for each of the three clusters were uploaded to the Immgen dataset and visualized in heatmaps as displayed in **Figure 2C** and **Supplementary Figure 1A** (http://rstats.immgen.org/MyGeneSet_New/index.html). Assignments were cross-checked by uploading this gene list to Enrichr (https://maayanlab.cloud/Enrichr/) and comparing the gene signatures to the scRNA-seq signature-based cell assignment database for mouse and human scRNA-seq, PanglaoDB Augmented 2021 (28).

### IgL identification with degenerated primers

High-affinity IBCs were magnetically enriched from VH125.C57BL/6-H2g7 splenocytes and 1,000 of these cells were bulk cell-sorted, spun down, and lysed in Trizol (Invitrogen). After total RNA extraction with Trizol according to manufacturer’s protocol, cDNA was synthesized using SuperScript IV reverse transcriptase (Invitrogen) according to manufacturer’s protocol. Variable regions of light chains were amplified using degenerated primers, previously characterized (11, 29). Briefly degenerate murine primers: forward 5′-ATT GTK MTS ACM CAR TCT CCA-3’ and reverse 5′-GGA TAC AGT TGG TGC AGC ATC-3’ (K substitutes G or T, M - A or C, R - A or G, S - C or G) were used to amplify light chain variable regions. PCR products were TA-cloned into pGEM^®^-T Easy Vector (Promega) overnight at 4°C, and ligation products were transformed into chemically competent DH5α *E.coli* (prepared in the Cambier lab). Sequencing was performed directly from colonies (Eton Bioscience) with forward T7 and reverse SP6 primers recognizing flanks near multiple cloning site of pGEM^®^-T Easy Vector. Variable genes were identified using IgBlast (http://www.ncbi.nlm.nih.gov/igblast/).

### Generation of recombinant antibodies (recAbs)

Variable regions of light chains were synthesized (Gene Universal). Next, the synthesized variable region genes of VH125 and light chains were cloned into human Igγ1 (the NCBI GenBank accession numbers: FJ475055) and Igκ (the NCBI GenBank accession numbers: FJ475056) expression vectors, respectively, as previously described (30). Human embryonic kidney (HEK) 293 cells were cultured in advanced DMEM with 1% L-glutamine (Gibco) and 10% low-IgG FBS (prepared in the Cambier lab). Expression vectors containing light chain and VH125 were co-transfected into HEK293 using polyethylenimine. Four hours post transfection medium was exchanged to Pfhm II protein free hybridoma medium (Gibco). Supernatants containing recombinant antibodies (recAbs) were collected on day 3 and 6 post-transfection. 50ml of supernatant was typically collected from transfected cells, spun down to remove cell debris, and incubated with 150µl recombinant protein A agarose beads (Genesee Scientific) overnight at 4°C on a rocker. Next, agarose beads were spun down, loaded on a column, and washed with 1M NaCl and PBS, and eluted with 200µl 0.1M glycine pH 3.0 for 10min on a rocker at room temperature and neutralized with 15μl 1M Tris HCl pH 8.0,. The eluted antibodies were dialyzed in 7000 MWCO 0.1-0.5ml dialysis cassette (Thermo Scientific) overnight at 4°C in a 4L PBS. 200-500μg of antibodies were usually collected and purity determined by SDS-PAGE and Coomassie blue staining. Concentration of antibodies was determined with NanoDrop™ One (Thermo Scientific).

### Enzyme-linked immunosorbent assay (ELISA)

ELISA microtiter plates (Costar) were coated with 10μg/ml recombinant human insulin (Sigma-Aldrich) in PBS overnight at 4°C. Plates were then washed with 0.5% Tween-20/PBS washing buffer and blocked using 1.5% cold fish gelatin (Sigma)/1mM EDTA/PBS blocking buffer for 90min at 37°C. Plates were washed between all subsequent steps three times in washing buffer for 5min at 37°C. Dialyzed recAbs were diluted in blocking buffer, loaded into plate, serially diluted 1:1 for 7 dilutions in blocking buffer, and incubated for 1 hour at 37°C. Antibody binding was detected by horseradish peroxidase conjugated goat anti-mouse IgG (SouthernBiotech) at a dilution of 1:10,000 in blocking buffer for 1 hour at 37°C. Reactions were developed with 3,3′,5,5′-tetramethylbenzidine (TMB) (Invitrogen) for 10min followed with 1M HCl (Sigma-Aldrich). Optical density at 450nm was determined using a VERSAMax plate reader (Molecular Devices), and data were analyzed with SoftMax Pro6 software.

### Affinity measurements by surface plasmon resonance (SPR)

IBC-derived recAbs were tested for affinity for insulin on Biacore™ 3000 (GE Healthcare). Purified recAbs were bound to Biacore™ Sensor Chip Protein A, according to the manufacturer’s protocol (GE Healthcare). Briefly, 1X HBS-EP buffer (Fisher Scientific) was run over a protein A sensor chip at rate 10µl/min. RecAbs diluted to 25µg/ml were injected at flow rate 10 µl/min and with 12-15µl to yield approximately 2000 response units (RU). Next, 10µl of 1X HBS-EP buffer (Fisher Scientific) were injected to remove any unbound material. Serial dilutions (100µM, 20µM, 4µM, 800nM, 160nM, and 32nM) of recombinant human insulin in PBS at pH 7.2 (Sigma) were flowed over recAbs immobilized on chip. Association of insulin occurred for 1 minute and dissociation was 15 minutes total. Highest to lowest concentrations of insulin were tested for each recAb and the chip was completely stripped between insulin injections with 20µl 100mM glycine pH 1.5. Each insulin concentration was repeated. Analysis was performed with Scrubber2 software (BioLogic Software, Australia). Results were fitted into Langmuir model, and kinetic parameters: association rate constant (*k*a), dissociation rate constant (*k*d), equilibrium dissociation constant (*K*D=*k*d/*k*a) were calculated based on different insulin concentrations. Background and injection spike due to fluctuation in fluidics were omitted, insulin injection time was set to zero, and RU were normalized to frequency for graphing.

### Statistics

Statistical analysis was performed with one-way analysis of variance (ANOVA) followed by Tukey’s multiple comparisons test, Student’s t-test, and Mann-Whitney nonparametric t test. Figure legends include information about statistical analyses performed. Prism software version 8.0 (GraphPad Software, La Jolla, CA, USA) was used in statistical analyzes. Data were represented as the standard error of the mean (SEM).

## Results

### Prevalent surface expression of immunoglobulin kappa (Igκ) light chain in activated and anergic high-affinity insulin-reactive B cells (IBCs)

**Figure 1:**
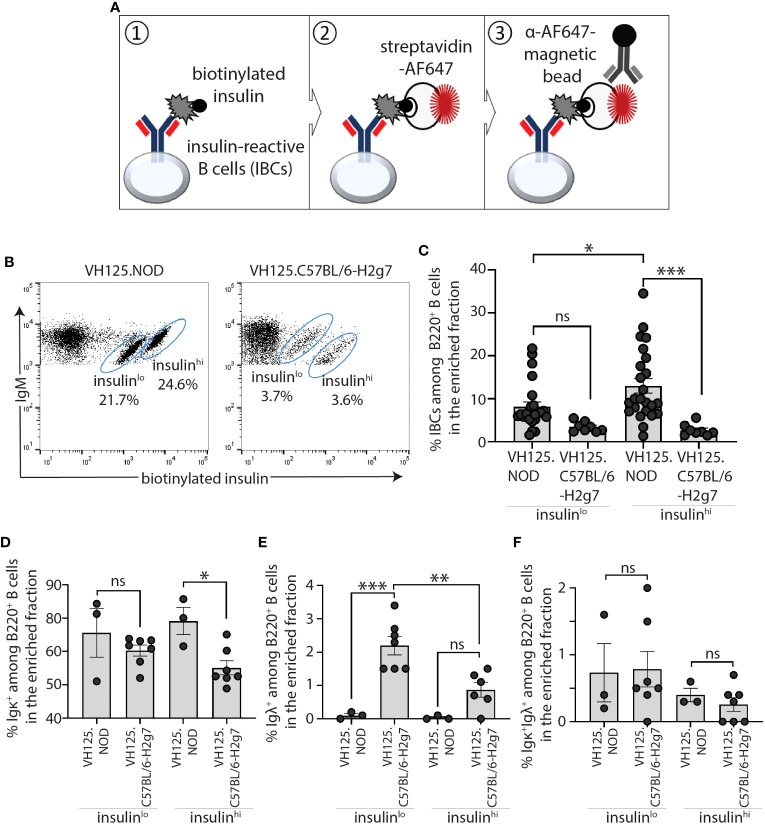
IBCs expressing Igκ accumulated in spleen of prediabetic VH125.NOD compared to diabetes-resistant VH125.C57BL/6-H2g7 mice. **(A)** Diagram representing magnetic bead-based methodology for enrichment of IBCs. **(B, C)** Frequency of splenic IBCs in the enriched fraction in prediabetic VH125.NOD and diabetes-resistant VH125.C57BL/6-H2g7: representative flow cytometric panels of IBCs gated on lymphocytes/singlets/live cells/B220^+^IgM^+^ cells are shown **(B)** and frequencies of the two IBC populations with insulin MFI low (insulin^lo^) and high (insulin^hi^) across multiple experiments are shown (VH125.NOD – n=24, VH125.C57BL/6-H2g7 – n=8) **(C)**. **(D–F)** Cell surface expression of immunoglobulin kappa (Igκ^+^) **(D)**, lambda (Igλ^+^) **(E)**, and double positive kappa and lambda (Igκ^+^Igλ^+^) **(F)** on insulin^lo^ and insulin^hi^ IBCs in VH125.NOD (n=3) and VH125.C57BL/6-H2g7 (n=7). Results are pooled from at least three independent experiments and represented as the mean ± SEM. Each dot represents a mouse. One-way ANOVA followed by *post hoc* Tukey’s multiple comparisons tests for panels **(C–F)** were used to determine the significance of differences among groups and defined as *P < 0.05, **P < 0.005, ***P < 0.0005. ns, no statistical significance.

**Figure 2:**
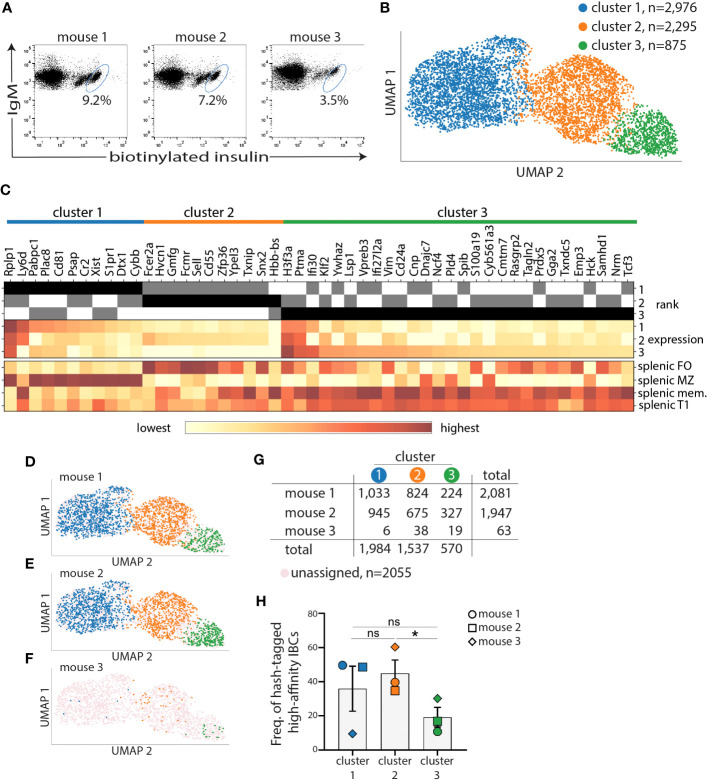
Marginal zone and follicular B-cell transcriptional clusters encompass most of high-affinity IBCs of VH125.NOD. Splenocytes from three prediabetic VH125.NOD were labeled with three different hashtag antibodies, cell sorted for high-affinity IBCs, pooled, and subjected to scRNA-seq on the 10X Genomics platform. **(A)** Flow panels representing sorted high-affinity IBCs (gated on lymphocytes/singlets/live cells/B220^+^IgM^+^ cells) from three prediabetic VH125.NOD mice. **(B)** Three clusters identified among high-affinity IBCs by unsupervised 10X CellRanger clustering method (blue – cluster 1, orange – cluster 2, green – cluster 3). **(C)** Heatmap of fifty differentially expressed genes among clusters. High-affinity IBCs from each cluster and donor mouse, overlayed on UMAP **(D–F)**, with cell numbers for each cluster, including unassigned cells, **(G)**, and frequency **(H)** (each symbol represents the same mouse as in **Figure 3**: circle – mouse 1, square – mouse 2, diamond – mouse 3). One-way ANOVA, followed by *post hoc* Tukey’s multiple comparisons tests were used to determine the significance of differences among groups. *P < 0.05, ns, no statistical significance.

As IBCs represent only 1-2% and < 0.5% of splenic B cells in prediabetic VH125.NOD and VH125.C57BL/6-H2g7, respectively, we adapted a previously described approach to enrich IBCs prior to scRNA-seq analysis (23). Briefly, this involved sequential staining of ex vivo splenocytes with biotinylated human insulin followed by streptavidin-fluorophore AF647 and anti-AF647 antibody-conjugated magnetic beads for the IBC isolation on a magnetic column (**Figure 1A**). Consistent with our previous reports and those of others, the procedure resolved a nonspecific “passenger” population and two insulin-binding populations with lower (insulin^lo^) and higher (insulin^hi^) binding of biotinylated-insulin-AF647 which laid on distinct diagonals relative to mIgM level (**Figure 1B**) (22, 23). Akin to our previous report, the frequencies insulin^hi^ IBCs differed between two mouse strains (**Figure 1C**) (23). To define the repertoire of light chains used by B cell in these populations, we analyzed the surface expression of immunoglobulin kappa (Igκ) and lambda (Igλ) among insulin^lo^ and insulin^hi^ IBCs (**Figures 1D–F**). Similar to our previous findings, Igκ expressing cells were found in both VH125.NOD and VH125.C57BL/6-H2g7 but were at significantly higher frequency in VH125.NOD (**Figure 1D**) (23). Igλ cells were virtually undetectable among insulin^lo^ and insulin^hi^ IBCs of VH125.NOD, likely indicative of inefficient central tolerance in NOD mice (**Figure 1E**). Although B cells expressing both Igκ and Igλ reportedly contribute to autoreactivity, we did not observe significant differences in dual expressors among IBC populations (31) (**Figure 1F**). It has been previously shown by the phenotypic analysis of B cell subsets and of receptor editing that insulin^lo^ and insulin^hi^ IBCs are of low and high affinity, and that the insulin^hi^ IBCs of VH125.NOD were activated, respectivey, indicated by IgM upregulation compared to non-specific “passenger” B cell population, and that their counterparts of VH125.C57BL/6-H2g7 were anergic, indicated by IgM downregulation, respectively (22, 23). Furthermore, our cytograms of insulin binding vs IgM (**Figure 2B**), the insulin^lo^ and insulin^hi^ populations are found on two different diagonals. These diagonals have distinct ratios of IgM expression to insulin binding and as such indicate different receptor affinity. Thus, we anticipate that insulin^lo^ and insulin^hi^ IBCs are of low and high affinity, respectively. To conclude, our flow cytometric analysis of IBCs confirmed higher frequency of IBCs and predominant expression of Igκ in prediabetic VH125.NOD.

### Follicular, marginal zone, and memory/T1 phenotypes among high-affinity IBCs from prediabetic VH125.NOD revealed by transcriptional profiling

We next sought to characterize the BCR repertoire and transcriptome of high-affinity IBCs from prediabetic VH125.NOD *via* scRNA-seq. Splenocytes of each of three donor mice were barcoded with anti-MHC antibody-conjugated to unique oligonucleotide tags, termed hashtags. Such barcoding enables tracing of each cell and its transcriptome to a specific donor. Following IBCs enrichment, we sorted by FACS 20080, 16105, and 818 high-affinity IBCs from three prediabetic VH125.NOD female mice which were barcoded with hashtag 1, 2, and 3, respectively (**Figure 2A**). The lower number of high-affinity IBCs of enriched B cells obtained from mouse 3 likely stems from a lower enrichment recovery of 3.5% in mouse 3 compared to 9.2% and 7.2% in mouse 1 and mouse 2, respectively (**Figure 2A**). In this and in our previous report, we observed that there is a recovery range of enriched high-affinity IBCs in VH125.NOD mice using our enrichment method (**Figure 1C**) (23). Although the three mice were siblings, same age and prediabetic status, mouse 3 was not housed together with mouse 1 and 2. Additionally, food and water intake were not controlled in this experiment. Whether the above factors impact IBC number will require further studies. Still, the lower cell recovery for mouse 3 limited the subsequent analysis.

Next, we pooled the sorted cells into one sample for droplet-encapsulation, cDNA synthesis, and library construction using the 10x Genomics protocols. We generated three libraries from the pool: 5’ total gene expression to represent cellular transcriptome, VDJ to reveal BCR repertoire, and hashtag to track down each transcript and BCR to one of the three donor mice.

We used the 10x Cell Ranger pipeline to process raw sequencing reads and 10x Loupe Cell Browser platform with Uniform Manifold Approximation and Projection (UMAP) algorithm for dimension reduction and data visualization. In UMAP, each cell is represented by a dot and the distance between each dot corresponds to distance within multi-dimensional transcriptional space. As such, dots corresponding to cells with similar transcriptional signatures are closer together, whereas dots corresponding to cells with more divergent transcriptional profiles are further apart. We identified three clusters: cluster 1 (blue, comprised of 2,976 cells), cluster 2 (orange, 2,295 cells), and cluster 3 (green, 875 cells) among high-affinity IBCs combined from three prediabetic VH125.NOD mice (**Figure 2B**). We restricted our analysis to the 52 genes with significantly differentiated expression between the three clusters. To assign the high-affinity IBCs to previously defined B cell subpopulations, we compared the 52 genes to transcriptional signatures of different B cell subsets deposited in the Immunological Genome Project (ImmGen). Out of the 52 differentially expressed genes, 50 genes were mapped to the ImmGen cell atlas. Genes overrepresented in cluster 1 and cluster 2 were enriched for those specific to splenic marginal zone (MZ) and follicular (FO) B cells, respectively (**Figure 2C**, **Supplementary Figure 1A**). Of note, cluster 2 genes were additionally over-represented in transitional T3 cells (**Supplementary Figure 1A**). The genes overrepresented in cluster 3 were similar to memory B and splenic T1 cells (**Figure 2C**, **Supplementary Figure 1B**).

Next, we used hashtag-barcodes to trace individual high-affinity IBCs to the mouse of origin and displayed the contribution of mouse 1, 2, 3 to each of the three clusters (**Figures 2D–G**). Cells that were insufficiently hashtagged were grouped as unassigned. The hashtag library was dominated by high-affinity IBCs from mouse 1 and mouse 2 with 2081 and 1947 cells, respectively, and represented by only 63 cells from mouse 3 (**Figure 2G**). We observed no statistically significant difference in the frequency of high-affinity IBCs between cluster 1 and 2 (**Figure 2H**). However, we observed a skewing of distribution when comparing individual mice and cell cluster assignments (**Figure 2G**, **Supplementary Figure 1C**). The low cell number recovered from mouse 3 is likely impacting the spread in the data. Overall, our data indicate that the transcriptome of high-affinity IBCs is consistently diversified across at least three clusters in prediabetic VH125.NOD mice.

### High-affinity IBCs of prediabetic VH125.NOD express a skewed light chain repertoire

**Figure 3:**
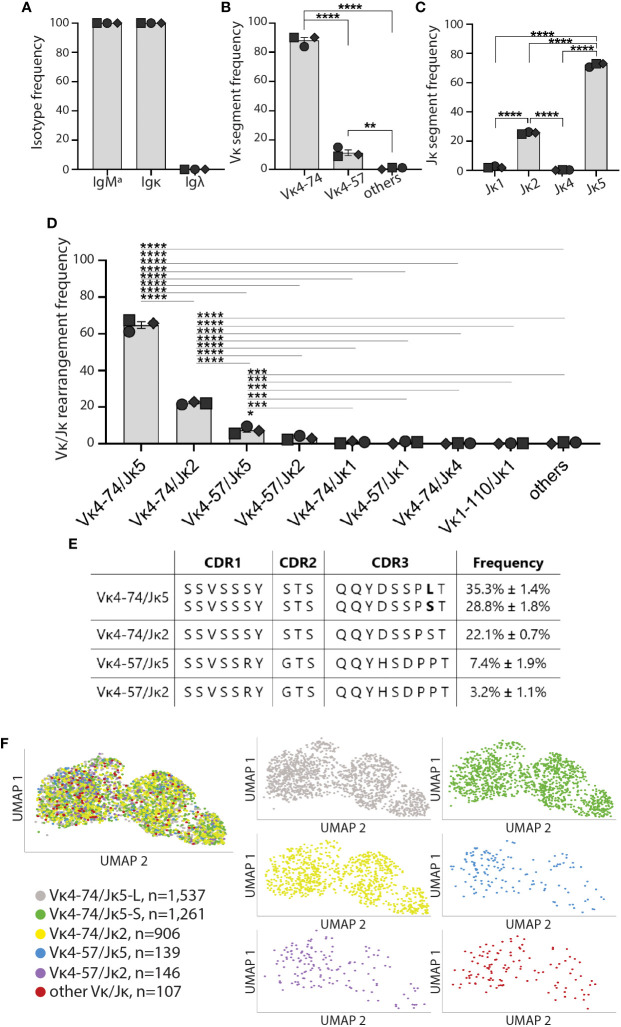
High-affinity IBCs of VH125.NOD expressed a biased Igκ repertoire. High-affinity IBCs from three prediabetic VH125.NOD mice from **Figure 2** were additionally subjected to VDJ sequencing. **(A)** Heavy and light chain isotype frequency, **(B)** Vκ gene segment frequency, **(C)** Jκ gene segment frequency, **(D)** Vκ/Jκ gene rearrangement frequency among high-affinity IBCs (each symbol represents a mouse: circle – mouse 1, square – mouse 2, diamond – mouse 3). **(E)** Amino-acid composition in complementarity determining region (CDR) 1, 2, and 3 in the five predominant light chains among high-affinity IBCs. **(F)** Distribution of five predominant light chains on the UMAP gene expression (different colors representing five predominant light chains, other Vκ/Jκ rearrangements were grouped and designated as “other Vκ/Jκ”). One-way ANOVA, followed by *post hoc* Tukey’s multiple comparisons tests were used to determine the significance of differences among groups. *P < 0.05, **P < 0.005, ***P < 0.0005, ****P < 0.00005.

We analyzed the heavy and light chain repertoire of high-affinity IBCs from the three prediabetic VH125.NOD. All 4,096 sequenced high-affinity IBCs expressed the same heavy chain, and its sequence alignment to the IgM^a^ VH125 heavy chain transgene revealed no differences in amino acid composition in the CDRs (**Supplementary Table 1**) (32). Our result is consistent with the previous observations that the IgM^a^ VH125 transgene allelically excludes the endogenous IgM^b^ heavy chain (**Figure 3A**) (15, 33). Transcriptionally, high-affinity IBCs expressed only Igκ chain (**Figure 3A**). Of note, we did not find a single cell expressing Igλ chain transcript among examined 4,096 high-affinity IBCs which is congruent with our staining data (**Figure 3A** and **Figure 1E**). Next, we analyzed the distribution of Vκ and Jκ gene segments separately and in rearrangements (**Figures 3B–D**). High-affinity IBCs preferentially expressed Vκ4-74 (87.9% ± 3.6%) and Jκ5 gene segments (72.2% ± 1.3%) (**Figures 3B, C**). Consequently, the predominant Vκ/Jκ rearrangement among high-affinity IBCs was Vκ4-74/Jκ5 (64.1% ± 3.1%), followed by Vκ4-74/Jκ2 (22.1% ± 0.7%), Vκ4-57/Jκ5 (7.4% ± 1.9%), and Vκ4-57/Jκ2 (3.2% ± 1.1%) (**Figure 3D**). Other Vκ/Jκ combinations occurred at the frequency less than 0.2% and were grouped as “others” (**Supplementary Figure 2**). Interestingly, the predominant Vκ4-74/Jκ5 encoded a CDR3 with either leucine (L, 35.3% ± 1.4%) or serine (P, 28.8% ± 1.8%) at amino acid position 97 which falls at the joint of Vκ and Jκ segments (**Figure 3E**). The occurrence of either L or S likely arises by addition and/or removal of nucleotides at this site during BCR rearrangement in B cell development. Importantly, none of the variable regions contained somatic mutations in their CDRs (11, 26). Lastly, we overlayed the dominant Vκ/Jκ light chain rearrangements on the UMAP gene expression to test whether either of them is associated with MZ, FO, or memory/T1 B cells identified in **Figures 2B, C**. We observed that the rearrangements distributed equally among three transcriptional clusters (**Figure 3F**, **Supplementary Figure 3**). In summary, high-affinity IBCs of prediabetic VH125.NOD mice expressed Igκ repertoire of low diversity and exclusivity of transcriptional MZ, FO, or memory/T1 profile.

### Unmutated and polymorphic Igκ is recovered from high-affinity IBCs of diabetes-resistant VH125.C57BL/6-H2g7

**Figure 4:**
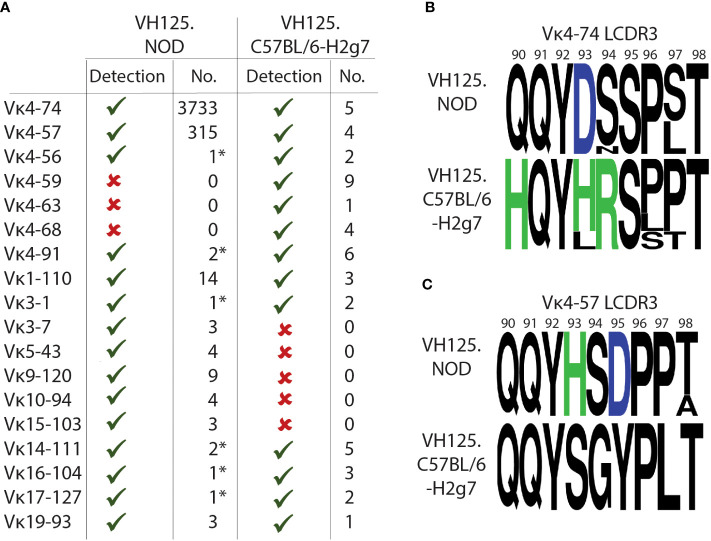
High-affinity IBCs of VH125.C57BL/6-H2g7 express unmutated and polymorphic Igκ chains. **(A)** Comparison of Vκ gene segments from splenic high-affinity IBCs of prediabetic VH125.NOD with degenerate primers (denoted by asterisk *) and by scRNA-seq as well as of VH125.C57BL/6-H2g7 with degenerate primers. Vκ presence (green check mark), absence (red cross) and the number of unique sequences detected are provided. **(B, C)** Amino acid composition of Igκ CDR3 for Vκ4-74 **(B)** and Vκ4-57 **(C)** in high-affinity IBCs of prediabetic VH125.NOD and VH125.C57BL/6-H2g7. Neutral amino acids marked in black, positively charged H (histidine) and R (arginine) in green, negatively charged D (aspartic acid) in blue.

We next sought to compare Igκ chain gene usage by anergic high-affinity IBCs from diabetes-resistant VH125.C57BL/6-H2g7. We conducted IBC enrichment followed by purification of high-affinity IBCs by FACS sorting as described above. Sorted cells from multiple diabetes-resistant VH125.C57BL/6-H2g7 female mice were pooled. Igκ chain representation was assessed by PCR using previously characterized degenerate primers (11, 26). Although this approach cannot be used to estimate frequencies of cells expressing specific Vκ/Jκ rearrangements, it is helpful to establish their representation. Vκ4-74 and Vκ4-57 were found among high-affinity IBCs of diabetes-resistant VH125.C57BL/6-H2g7 similar to their counterparts in prediabetic VH125.NOD (**Figure 4A**). Other Vκ gene segments such as Vκ4-56, Vκ4-91, Vκ1-110, Vκ3-1, Vκ14-111, Vκ16-104, Vκ17-127, Vκ19-93 were also represented among high-affinity IBCs of both genetic backgrounds (**Figure 4A**). However, certain Vκ gene segments were unique to either high-affinity IBCs of prediabetic VH125.NOD (Vκ3-7, Vκ5-43, Vκ9-120, Vκ10-94, Vκ15-103) or diabetes-resistant VH125.C57BL/6-H2g7 (Vκ4-59, Vκ4-63, Vκ4-68) (**Figure 4A**).

We observed amino acid differences in the Vκ/Jκ rearrangements containing Vκ4-74 and Vκ4-57 from high-affinity IBCs of prediabetic VH125.NOD and diabetes-resistant VH125.C57BL/6-H2g7 (**Figures 4B, C**). Glutamine at position 91 (Q91) and tyrosine at position 92 (Y92) were conserved in Vκ4-74- and Vκ4-57-containing rearrangements in both genetic backgrounds. Furthermore, our observations are consistent with the previous report of amino acid polymorphism differences in Igκ in VH125.NOD and VH125.C57BL/6-H2g7 backgrounds (11). Interestingly, some substitutions in the Vκ4-74-rearrangment involved exchanges of negatively charged aspartic acid (D93) and neutral serine (S94) in VH125.NOD into positively charged histidine (H93) and arginine (R94) in VH125.C57BL/6-H2g7. In contrast, the Vκ4-57-containing rearrangement from VH125.NOD harbored a positively charged H93 and negatively charged D95 which were substituted into neutral S93 and Y95 in VH125.C57BL/6-H2g7. As insulin carries an overall negative charge, amino-acid polymorphism with an altered charge may affect the binding. Interestingly, the amino acid substitutions to negatively charged D in the CDR2 of VH125 improved insulin recognition suggesting that instead of direct interactions with insulin those substitutions may enhance binding by alternating the binding site structure (32). In summary, the Igκ of high-affinity IBCs of VH125.C57BL/6-H2g7 contained the same Vκ/Jκ rearrangements as seen in VH125.NOD, but with amino acid differences attributable to polymorphisms.

### Independent of polymorphism Vκ4-74-containing recAbs bind insulin with high affinity

**Figure 5:**
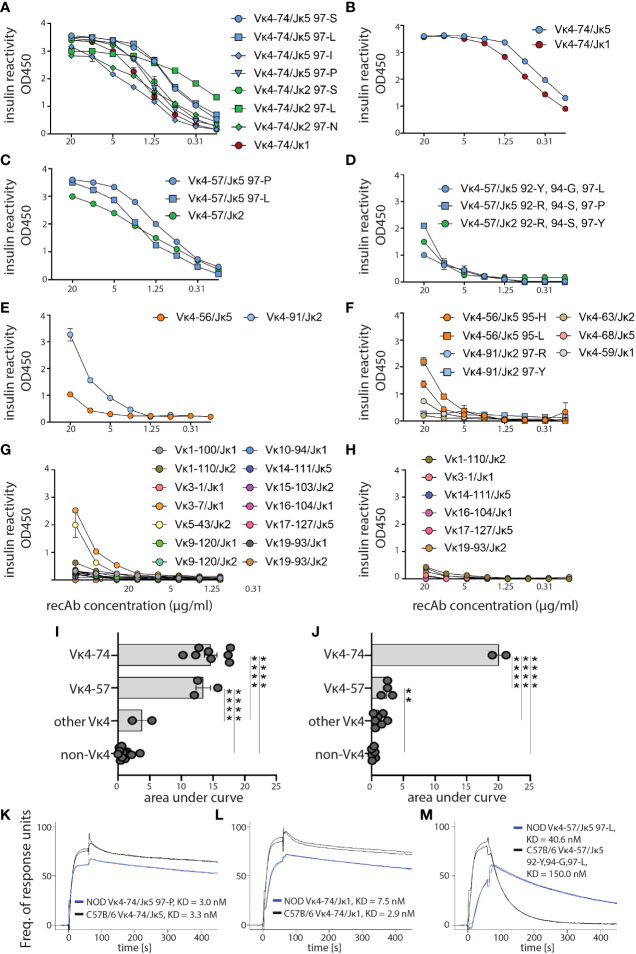
Affinity of anti-insulin binding antibodies from VH125.NOD and VH125.C57BL/6-H2g7 genetic backgrounds. Igκ chains from splenic high-affinity IBCs of VH125.NOD and VH125.C57BL/6-H2g7 by scRNA-seq and sequencing with degenerate primers, respectively, were selected for recombinant antibody (recAb) expression. **(A–H)** ELISA with insulin coated plates for specificity measurements with recAbs containing VH125 heavy chain and light chains derived from splenic high-affinity IBCs of either VH125.NOD **(A, C, E, G)** or VH125.C57BL/6-H2g7 **(B, D, F, H)**. RecAbs were grouped into Vκ4-74 **(A, B)**, Vκ4-57 **(C, D)**, other Vκ4 families **(E, F)**, and non-Vκ4 **(G, H)**. **(I, J)** Area under ELISA curves for different groups of recAbs containing Igκ of VH125.NOD **(I)** and VH125.C57BL/6-H2g7 **(J)**. **(K, L)** SPR curves indicating affinity (KD) and kinetics of 0.8µM insulin binding by recAbs with Vκ4-74 **(K, L)** and Vκ4-57 **(M)**. Results are pooled from at least three independent experiments and represented as the mean ± SEM. One-way ANOVA, followed by *post hoc* Tukey’s multiple comparisons tests. **P < 0.005, ****P < 0.00005.

To compare the specificity and affinity of high-affinity IBCs to insulin, we selected 45 light chains from high-affinity IBC of prediabetic VH125.NOD (27 candidates) and VH125.C57BL/6-H2g7 (18 candidates) for recombinant antibody (recAb) generation (**Supplementary Table 2**). Each of the recAbs contained one Igκ and VH125, and all were tested for reactivity against insulin coated on ELISA plates (**Figures 5A–J**). Vκ4-74-containing recAbs bound insulin independent of J segment usage, amino acid at 97 position, and polymorphic differences between NOD and C57BL/6 Igκs (**Figures 5A, B**). In contrast, Vκ4-57-containing recAbs of VH125.NOD bound insulin better than their VH125.C57BL/6-H2g7 counterparts (**Figures 5C, D**). Some of the recAbs containing other Vκs (Vκ4-56 and Vκ4-91) also recognized insulin (**Figures 5E, F**). We also recovered Igκs which did not belong to Vκ4 gene family (here designated as non-Vκ4 recAbs) in the scRNA-seq and bulk-sorting/degenerate primer approaches (**Figures 3**, **4**). Except for Vκ3-7/Jκ1 and Vκ5-43/Jκ2 of VH125.NOD genetic background, neither of non-Vκ4 recAbs recognized insulin suggesting that they were found in the high-affinity IBC population due to binding to non-insulin components of our enrichment methodology (**Figures 5G, H**). Next, we analyzed the kinetics of Vκ4-74- and Vκ4-57-containing recAbs binding to insulin by surface plasmon resonance where different concentrations of insulin were flowed over immobilized recAbs (**Figures 5K–M**). Interestingly, recAbs with Vκ4-74 derived from VH125.NOD and VH125.C57BL/6-H2g7 had comparable affinities to insulin with fast Kon and slow Koff (**Figures 5K, L**). In contrast, affinity of recAbs with Vκ4-57 from VH125.C57BL/6-H2g7 was lower compared to its counterpart in VH125.NOD (**Figure 5M**). To conclude, high-affinity IBCs express clonal BCRs with similar insulin binding affinities despite being rendered anergic in VH125.C57BL/6-H2g7 and activated in VH125.NOD mice.

## Discussion

Silenced by anergy in healthy humans and diabetes-resistant mice, high-affinity IBCs loose anergy in individuals with new-onset diabetes and in some of their first-degree relatives, and diabetes-susceptible VH125.NOD mice (13, 23). Intrinsic factors that compromise anergy include perturbations in the B cell receptor signaling pathway and other autoimmunity risk alleles (14, 34). It is unclear to what extent affinity of BCR for autoantigens, notably insulin, plays a role in B cell participation in autoimmune diabetes. Here, we used two murine strains: prediabetic VH125.NOD and diabetes-resistant VH125.C57BL/6-H2g7 to explore the contribution of B cell affinity to a pancreatic islet antigen, insulin, in disease. In agreement with a previous report, we found that high-affinity IBCs predominantly express immunoglobulin kappa (Igκ) (**Figure 1**) (23). The application of scRNA-seq revealed marginal zone (MZ), follicular (FO), and memory/T1 B cell transcriptional profiles in high-affinity IBCs of prediabetic VH125.NOD mice (**Figure 2**). Surprisingly, we observed that the Igκ repertoire of these cells was confined to five Vκ/Jκ rearrangement none of which segregated into MZ, FO, or memory/T1 B cell transcriptional clusters (**Figure 3**). In parallel, the analysis of the Igκ repertoire of VH125.C57BL/6-H2g7 revealed primarily Vκ/Jκ rearrangements shared with VH125.NOD mice but containing amino acid polymorphism in the CDR3 (**Figure 4**). Interestingly, insulin specificity and affinity of the recombinant antibodies (recAbs) was associated with the Vκ4 family, especially Vκ4-74, independent of the diabetes risk of the mouse strain (**Figure 5**). We applied two mouse models, magnetic cell separation, FACS, and scRNA-seq to characterize VDJ repertoire and transcriptome of high-affinity IBCs.

We applied magnetic cell separation and FACS to obtain high-quality transcripts and antibody sequences. This approach allowed us to enrich and purify relatively rare high-affinity IBCs and subject those cells to scRNA-seq to recover three libraries: VDJ library for BCR repertoire, 5’ total gene expression library for transcriptome profiling, and hashtag library for tracking cells to their donors. The recent developments of scRNA-seq enable juxtaposition of these three libraries to link each B cell to its transcriptome, BCR sequence and mouse donor. We previously applied a similar strategy to characterize SARS-CoV-2 antigen-binding B cells derived from COVID-19-infected people (35). Likewise, others utilized similar approaches to deeply characterize B cell subsets (36, 37). In this study, we identified three distinct transcriptional profiles in high-affinity IBCs of VH125.NOD prediabetic mice: cluster 1 containing MZ, cluster 2 – FO, and cluster 3 – a mixture of memory B and T1 cell gene signatures. Our transcriptional assignment is in agreement with the previous reports identifying high-affinity IBCs expressing either CD21 (encoded by Cr2) or CD23 (encoded by Fcer2a) which are typical markers for identification of FO and MZ B cells (22, 23). Surprisingly, the cluster 2 genes were also over-represented in transitional T3 cells expressing similar cell surface markers to FO B cells such as CD23 (**Supplementary Figure 1A**) (38). T3 cells were shown to be anergic, suggesting that at least some of high-affinity IBCs of prediabetic VH125.NOD still retain anergic phenotype (38). The limited sample size of high-affinity IBCs with T3 profile precludes determination of whether these cells express particular Vκ/Jκ rearrangements. Notably, as T3 and a subset of FO B cells were reported to be antigen experienced, it is likely cells in cluster 2 are insulin-experienced (38, 39). Cluster 3, containing transcriptional markers of memory B and T1 cells likely harbors cells secreting anti-insulin antibodies as previously detected by ELISA in serum of VH125.NOD (23). The limited sample size hinders a better distinction between memory B cell and T1 gene signatures in this dataset. However, the comparison to known scRNA-seq signatures using the Enrichr gene set enrichment platform suggests a greater similarity to a memory B cell population as compared to naïve B cells (**Supplementary Figure 1B**). Future studies recovering scRNA-seq in combination with CD21, CD23, IgM, and AA4.1 (CD93) and CITE-seq will enable simultaneous transcriptome and phenotyping of high-affinity IBCs (40). This more granular analysis will increase cluster resolution and differentiation between FO and T3 cells in cluster 2 as well as memory and T1 cells in cluster 3. Additionally, CITE-seq with antibodies specific to CD69 or CD86 will be valuable in revealing the activation status of IBCs from spleen, pancreatic lymph nodes and pancreas.

In parallel with transcriptional profiling, VDJ repertoire revealed that high-affinity IBCs express a repertoire represented by five Vκ/Jκ rearrangements: predominant Vκ4-74/Jκ5, followed by Vκ4-57/Jκ5 and three other Vκ/Jκ. Henry-Bonami et al., previously recovered Vκ4-74/Jκ5 and Vκ4-57/Jκ5 from splenic and bone marrow-derived IBCs suggesting that biased Vκ usage occurs even in the absence of exogenous insulin (11, 26). The skewed repertoire of high-affinity IBCs of prediabetic VH125.NOD mice towards Vκ4-74/Jκ5 maybe be a consequence of defects in central tolerance in NOD genetic background wherein autoreactive B cells are not sufficiently trimmed by receptor editing and clonally deleted (11). Alternatively, in contrast to anergic B cells with very short half-life, activated B cells, such as high-affinity IBCs in VH125.NOD, may stay alive in the periphery longer contributing to their higher numbers (41). Surprisingly, the skewed Vκ/Jκ repertoire of high-affinity IBCs distributed evenly among transcriptional profiles of MZ, FO, and memory/T1 B cells, suggesting that B cell expressing the same BCR may have different experience with antigen. Whether this phenomenon is due to the stochastic exposures to insulin during IBC development or to different trafficking history remains to be explored. Still, our results agree with the previous report demonstrating weakening in tolerance mechanisms in NOD mice (11).

The comparison of Vκ4-74/Jκ5, Vκ4-74/Jκ1, and Vκ4-57/Jκ5 from NOD and C57BL/6 revealed the presence of conserved amino acids in the CDR3 and polymorphism. The conserved amino acid such as tyrosine (Y) at the position 92 affects the shape of binding site, provides access to hydrogen-bonding residues, and its mutation to phenylalanine (F) prevents insulin binding in VH125/mutant A12 antibody (24, 42). Whether the polymorphic amino acid in the CDR3 region of the light chain affects the binding is unclear. Amino acid differences in Vκ4-74/Jκ5 and Vκ4-74/Jκ1 with the exchange of neutral (90Q, 94S) and negatively (93D) charged amino acids in VH125.NOD to positively charged (90H, 93H, 94R) in VH125.C57BL/6-H2g7 did not affect the kinetics of insulin binding (**Figures 4B**, **5A, B, K, L**). In contrast, VH125.NOD-derived Vκ4-57Jκ5 rearrangements had higher affinity for insulin than their counterparts derived from VH125.C57BL/6-H2g7 (**Figures 5C, D, M**). It is unclear whether positively charged 93H, negatively charged 95D, or the QQY … PPT motif in the Vκ4-57/Jκ5 from VH125.NOD contribute to the enhanced affinity (11). In contrast to our findings, others reported that light chains with negatively charged D can reduce binding to self-antigens such as DNA (43, 44). Although Vκ4-57/Jκ5 constitutes only 7.4% of the repertoire of high-affinity IBCs of prediabetic NOD, the affinity of this receptor to insulin may be sufficient to drive B cell activation and their pathogenic activities. Indeed, Vκ4-57 light chain was found in single islets of VH125.NOD and is encoded in retrogenic VH125/A12 B cells that relocated to pancreatic lymph nodes following the adoptive transfer (24, 45). In the current study, we profiled Vκ repertoire of high-affinity IBCs from prediabetic VH125.NOD with scRNA-seq and diabetes-resistant VH125.C57BL/6-H2g7 with degenerate primer PCR. Future comparisons using scRNA-seq on both strains will enable direct comparison of B cell repertoire diversity, rearrangement frequencies and distribution among different B cell transcriptional clusters.

Interestingly, Vκ/Jκ rearrangements belonging to Vκ4 were associated with the specificity and high affinity to insulin. In this study, we analyzed the high-affinity IBCs derived from the spleen which are exposed to monomeric insulin. It is possible that the Vκ/Jκ rearrangements derived from the pancreas or pancreatic lymph nodes (PLN) would be associated with polymeric insulin that would more efficiently drive the B cell activation. Insulin in the form of insoluble crystalline hexamers is typically stored in beta cells in densely packed granules (46). Upon the damage of beta cells, these insulin hexamers are released and can potentially stimulate high-affinity IBCs migrating from the spleen and blood to the pancreas and PLN. In addition, the concentration of insulin is expected to be higher in the diabetic pancreas and PLN (40 × 10^-3^ M in beta cell granules) compared to blood (10 × 10^-10^ M) generating favorable conditions for loss of anergy and B cell activation in VH125.NOD mice (18, 46). It would be interesting to see whether the IBCs derived from the pancreas and PLN are associated with more insulin compared to those from spleen. Using scRNA-seq with the nucleotide-barcoded mAb123 which detects insulin associated with VH125-containing BCR, it would be possible to characterize the insulin occupancy on IBCs in relationship to their Vκ/Jκ repertoire and transcriptional profiles. In addition, our cell isolation protocol could be adapted to uniquely select cells bound to mono- versus polymeric insulin and compare characterize their phenotype and BCR affinity (47). Besides, investigations into the repertoire and gene expression signatures of low-affinity IBCs from both mouse strains would be of interest. Although we hypothesize that these cells are ignorant due to their low affinity BCR, their repertoire was reported to be distinct (11).

In summary, we used scRNA-seq to characterize high-affinity IBCs of prediabetic VH125.NOD, determining their Vκ/Jκ usage and transcriptional profile. BCR Ig light chains were found to be unmutated and largely restricted to Vκ4-74/Jκ5 and Vκ4-57/Jκ5. This was also the case for Vκ/Jκ for their anergic counterparts in diabetes-resistant VH125.C57BL/6-H2g7. While in VH125.NOD, these cells were found in “activated” MZ, FO, and memory B/T1 cell populations, in diabetes-resistant VH125.C57BL/6-H2g7 they were previously demonstrated to be anergic (23). Thus, in these strains B cell activation versus anergy does not track with divergent affinity for the autoantigen insulin. It is likely that despite their high-affinity BCRs IBCs of VH125.C57BL/6-H2g7 remain anergic due to intact tolerance mechanisms which silence BCR activation.

## Data availability statement

The scRNA-seq data presented in the study are deposited in the Gene Expression Omnibus (GEO) repository (https://www.ncbi.nlm.nih.gov/geo/), accession number GSE213973.

## Ethics statement

The animal study was reviewed and approved by the Institutional Review Board of the Research Compliance Office at the University of Colorado.

## Author contributions

Conceptualization: MB and JCC; experimentation: MB and AG; data analysis: MB, ITWH, and JCC; manuscript preparation: MB, ITWH, and JCC; project administration: MB, AG, and JCC; funding acquisition: MB, ITWH, AG, and JCC. All authors have read and agreed to the published version of the manuscript.

## Funding

Studies were funded by NIH Institutional T32 #5T32AI007405 (MB), Colorado Clinical and Translational Sciences Institute Pilot Grant Award #CO-M-21-51 (MB), R21 #1R21AI149019 (AG), R01 #5R01AI124487 (JCC), and Rheumatology Research Foundation Scientist Development Award (ITWH).

## Acknowledgments

We would like to express our gratitude for technical assistance to CU Anschutz Cancer Center Flow Cytometry Shared Resource, Genomics Shared Resource, Structural Biology and Biophysics Core Facilities as well as Bergren Crute, Bergren Crute, Nomin Javkhlan, Kenneth Link, and Makayla Windholz. We thank Drs. Roberta Pelanda, Raul Torres, and Mia Smith for reagents and helpful discussions. We would like to thank Soojin Kim for maintaining mouse colony.

## Conflict of interest

ITWH is partly funded by the Pfizer Global Grants Foundation Rheumatology program #51849703, but those funds did not support his work on this project.

The remaining authors declare that the research was conducted in the absence of any commercial or financial relationships that could be construed as a potential conflict of interest.

## Publisher’s note

All claims expressed in this article are solely those of the authors and do not necessarily represent those of their affiliated organizations, or those of the publisher, the editors and the reviewers. Any product that may be evaluated in this article, or claim that may be made by its manufacturer, is not guaranteed or endorsed by the publisher.

## Author disclaimer

The contents of this manuscript do not represent the views of the U.S. Department of Veterans Affairs or the United States Government.

## Supplementary material

Supplementary Figure 1 High-affinity IBCs of prediabetic VH125.NOD are characterized by marginal zone, follicular, and splenic memory B cell transcriptional profile. Splenocytes from three prediabetic VH125.NOD were labeled with three different barcoded antibodies, cell sorted for high-affinity IBCs, pooled, and subjected to single cell RNA sequencing on the 10X Genomics platform. **(A)** Heatmap of fifty differentially expressed genes among clusters and compared to different B cell subsets. **(B)** scRNA-seq signature analysis indicates a greater similarity of cluster 3 genes to memory B cell population as compared to naïve B cell population. Bar chart from comparison of cluster 3 genes to scRNA-seq signature database, PanglaoDB Augmented 2021, gene set analysis from Enrichr. The term “B Cell Memory” has the most significant overlap with the cluster 3 gene set. **(C)** Chi-squared (χ²) analysis of cell frequency by mouse and cluster assignment. Standard residuals (std. residuals) expressed as a ratio of the difference of the observed and expected values and the square root of the expected values. Normal distribution with mean 0 and standard deviation 1.

Supplementary Figure 2 Frequency of high-affinity IBCs of prediabetic VH125.NOD containing non-Vκ4 gene segments (circle – mouse 1, square – mouse 2, diamond – mouse 3).

Supplementary Figure 3 Unbiased distribution of five dominant Vκ/Jκ rearrangements among three transcriptional splenic B cell clusters. Three sequencing libraries: VDJ, transcriptome, and hashtagged from high-affinity IBCs of prediabetic VH125.NOD were overlayed to determine the assignment of each Vκ/Jκ to its mouse donor and transcriptional cluster on a single cell resolution. **(A)** Vκ4-74/Jκ5-L. **(B)** Vκ4-74/Jκ5-S. **(C)** Vκ4-74/Jκ2. **(D)** Vκ4-57/Jκ5. **(E)** Vκ4-57/Jκ2. **(F)** other Vκ/Jκ. Each symbol represents the same mouse as in and : circle – mouse 1, square – mouse 2, diamond – mouse 3. One-way ANOVA, followed by *post hoc* Tukey’s multiple comparisons tests were used to determine the significance of differences among groups.

Supplementary Table 1 Amino acid composition of the reference IgMa VH125 heavy chain transgene and heavy chains obtained from high-affinity IBCs by 10x scRNA-seq.

Supplementary Table 2 Amino acid composition of recombinant antibodies (recAbs) from VH125.NOD and VH125.C57BL/6-H2g7 genetic backgrounds. Amino acid composition of complementarity determining regions (CDR1-3) and framework 4 (FWR4) for recAbs composed of VH125 heavy chain and light chains derived from splenic IBCs of VH125.NOD (NOD) and VH125.C57BL/6-H2g7 (C57BL/6). Additional identification of amino acid differences in CDR3 is added for recAbs with the same Vκ/Jκ rearrangement.
