# Supplementary figures and images for: Comparative analysis of the repertoire of insulin-reactive B cells in type 1 diabetes-prone and resistant mice
Source: Front Immunol. 2022 Oct 4;13:961209. doi: 10.3389/fimmu.2022.961209 (PMC9579539; doi:10.3389/fimmu.2022.961209)

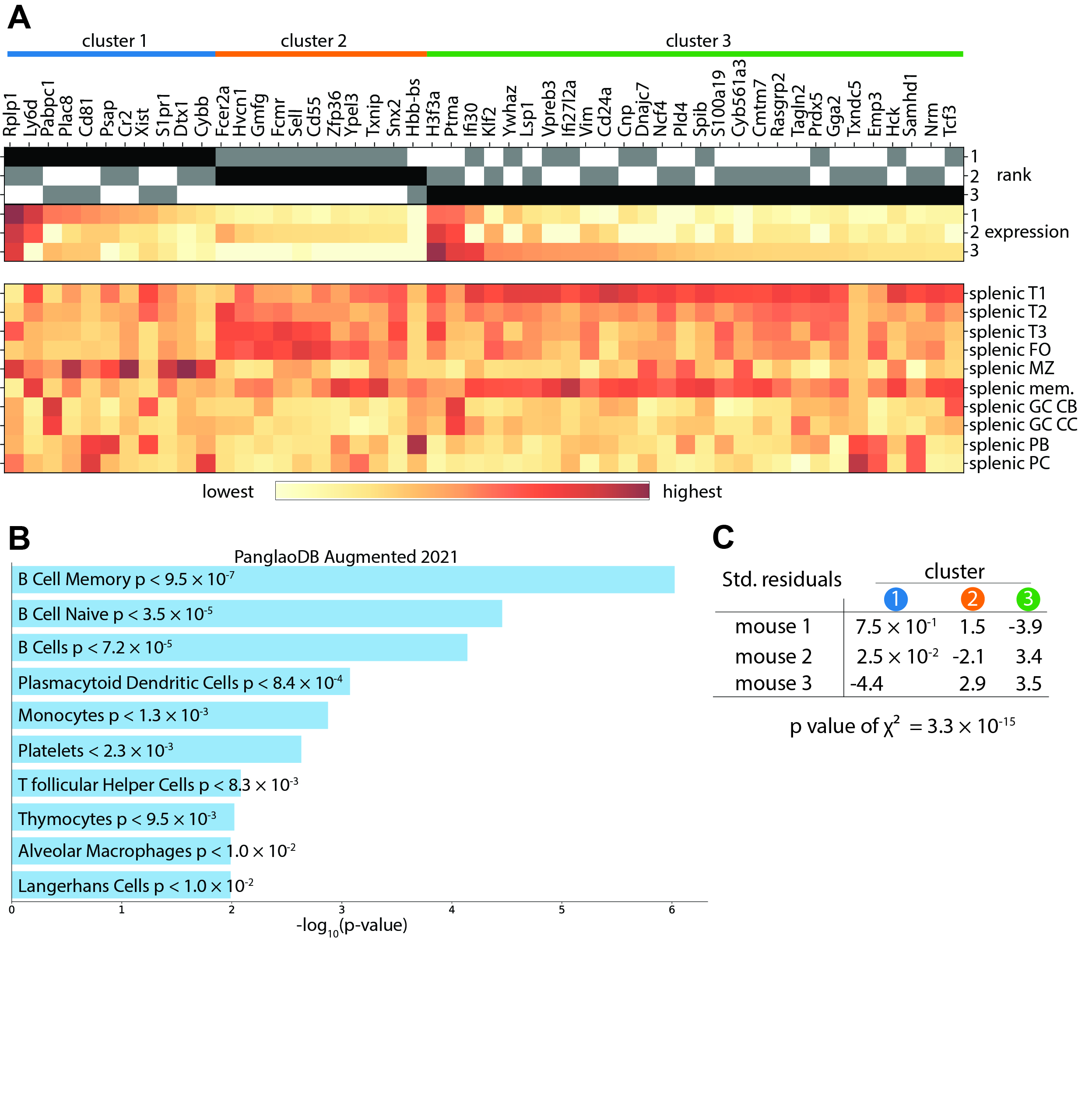

Supplement: Supplementary Figure 1 — High-affinity IBCs of prediabetic VH125.NOD are characterized by marginal zone, follicular, and splenic memory B cell transcriptional profile. Splenocytes from three prediabetic VH125.NOD were labeled with three different barcoded antibodies, cell sorted for high-affinity IBCs, pooled, and subjected to single cell RNA sequencing on the 10X Genomics platform. (A) Heatmap of fifty differentially expressed genes among clusters and compared to different B cell subsets. (B) scRNA-seq signature analysis indicates a greater similarity of cluster 3 genes to memory B cell population as compared to naïve B cell population. Bar chart from comparison of cluster 3 genes to scRNA-seq signature database, PanglaoDB Augmented 2021, gene set analysis from Enrichr. The term “B Cell Memory” has the most significant overlap with the cluster 3 gene set. (C) Chi-squared (χ²) analysis of cell frequency by mouse and cluster assignment. Standard residuals (std. residuals) expressed as a ratio of the difference of the observed and expected values and the square root of the expected values. Normal distribution with mean 0 and standard deviation 1. [file Image_1.jpg]

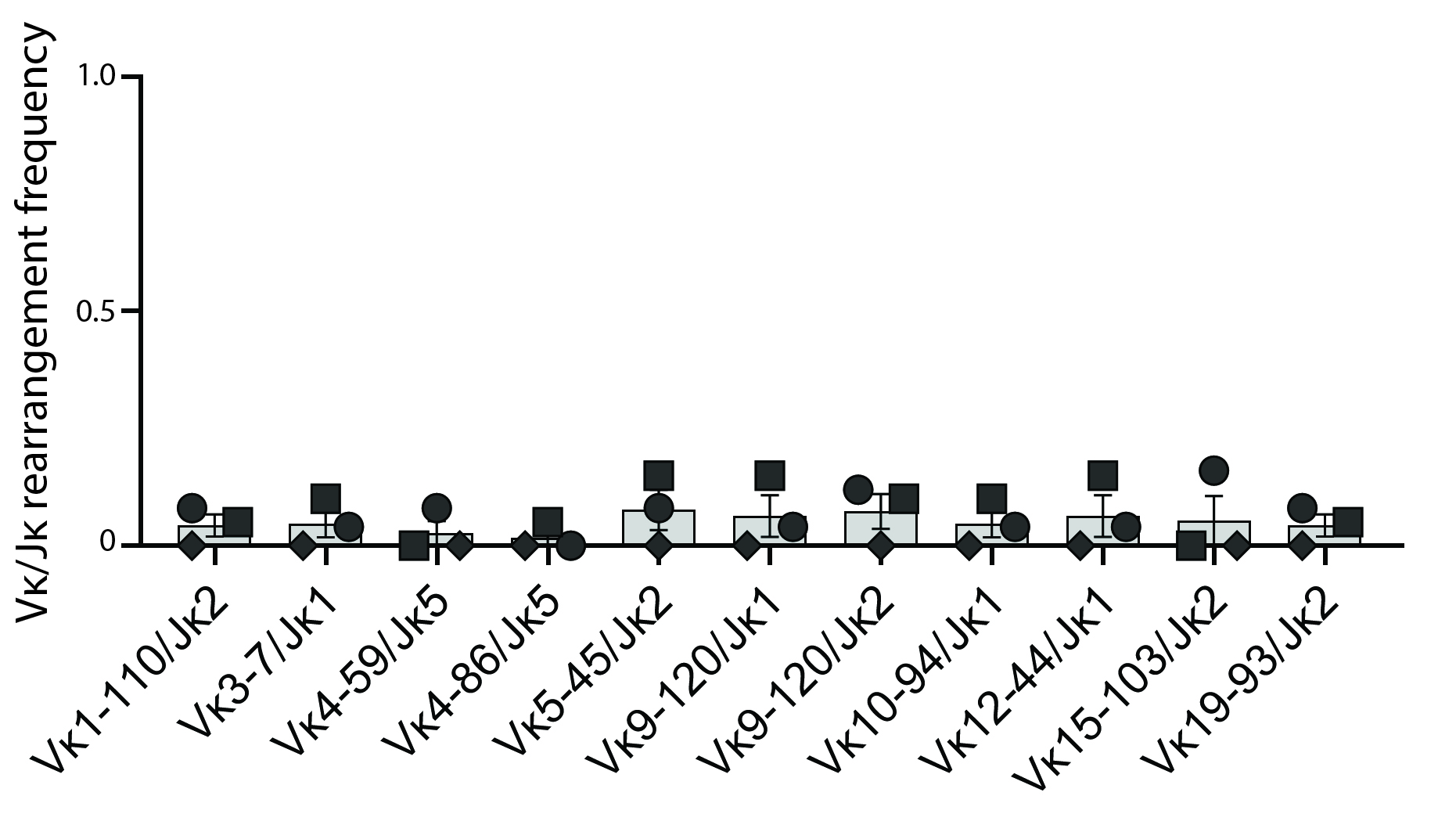

Supplement: Supplementary Figure 2 — Frequency of high-affinity IBCs of prediabetic VH125.NOD containing non-Vκ4 gene segments (circle – mouse 1, square – mouse 2, diamond – mouse 3). [file Image_2.jpg]

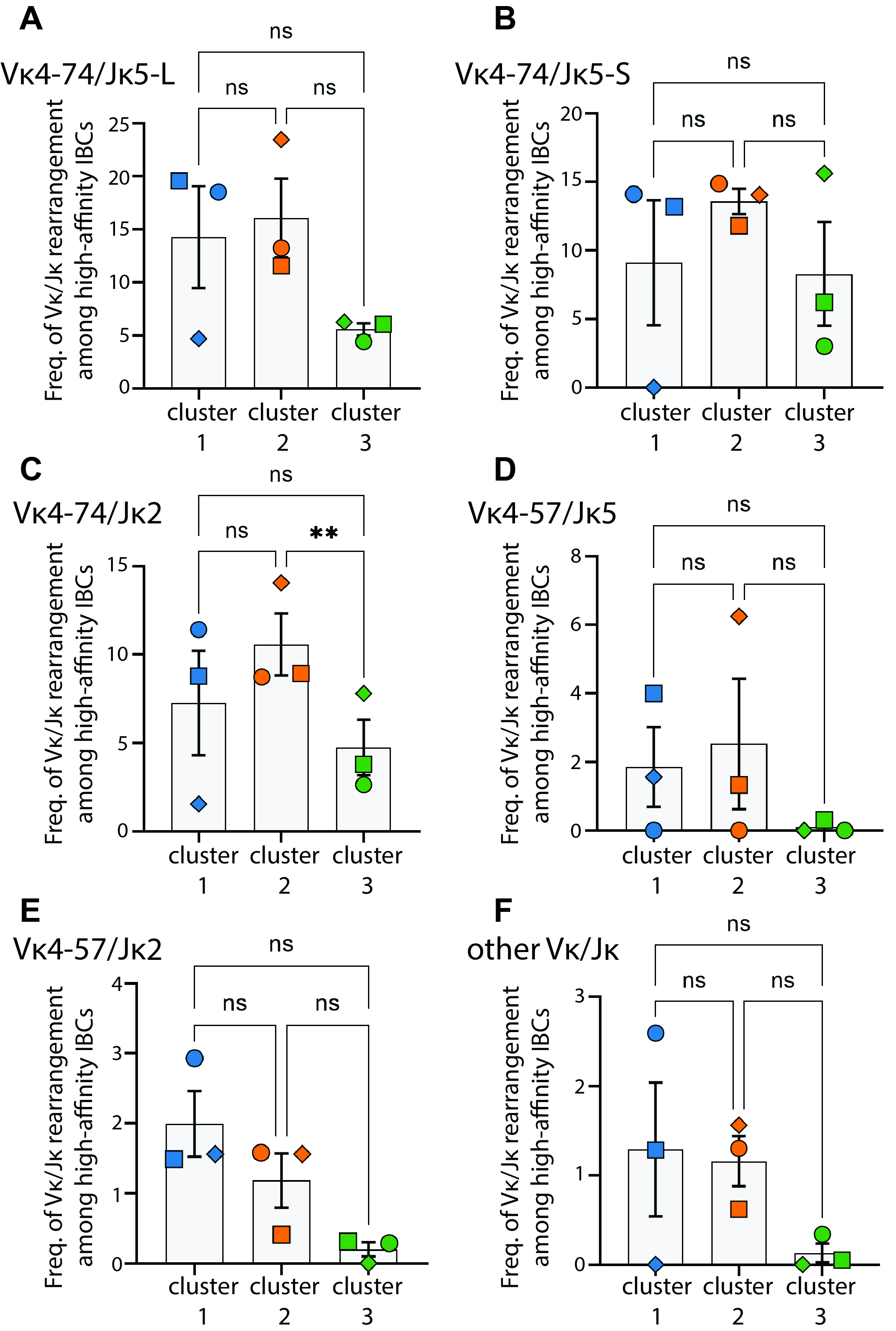

Supplement: Supplementary Figure 3 — Unbiased distribution of five dominant Vκ/Jκ rearrangements among three transcriptional splenic B cell clusters. Three sequencing libraries: VDJ, transcriptome, and hashtagged from high-affinity IBCs of prediabetic VH125.NOD were overlayed to determine the assignment of each Vκ/Jκ to its mouse donor and transcriptional cluster on a single cell resolution. (A) Vκ4-74/Jκ5-L. (B) Vκ4-74/Jκ5-S. (C) Vκ4-74/Jκ2. (D) Vκ4-57/Jκ5. (E) Vκ4-57/Jκ2. (F) other Vκ/Jκ. Each symbol represents the same mouse as in and : circle – mouse 1, square – mouse 2, diamond – mouse 3. One-way ANOVA, followed by post hoc Tukey’s multiple comparisons tests were used to determine the significance of differences among groups. [file Image_3.jpg]

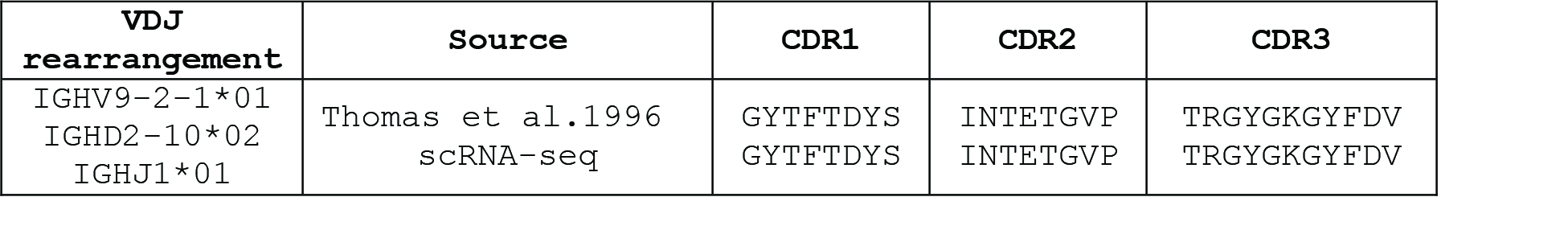

Supplement: Supplementary Table 1 — Amino acid composition of the reference IgMa VH125 heavy chain transgene and heavy chains obtained from high-affinity IBCs by 10x scRNA-seq. [file Image_4.jpg]

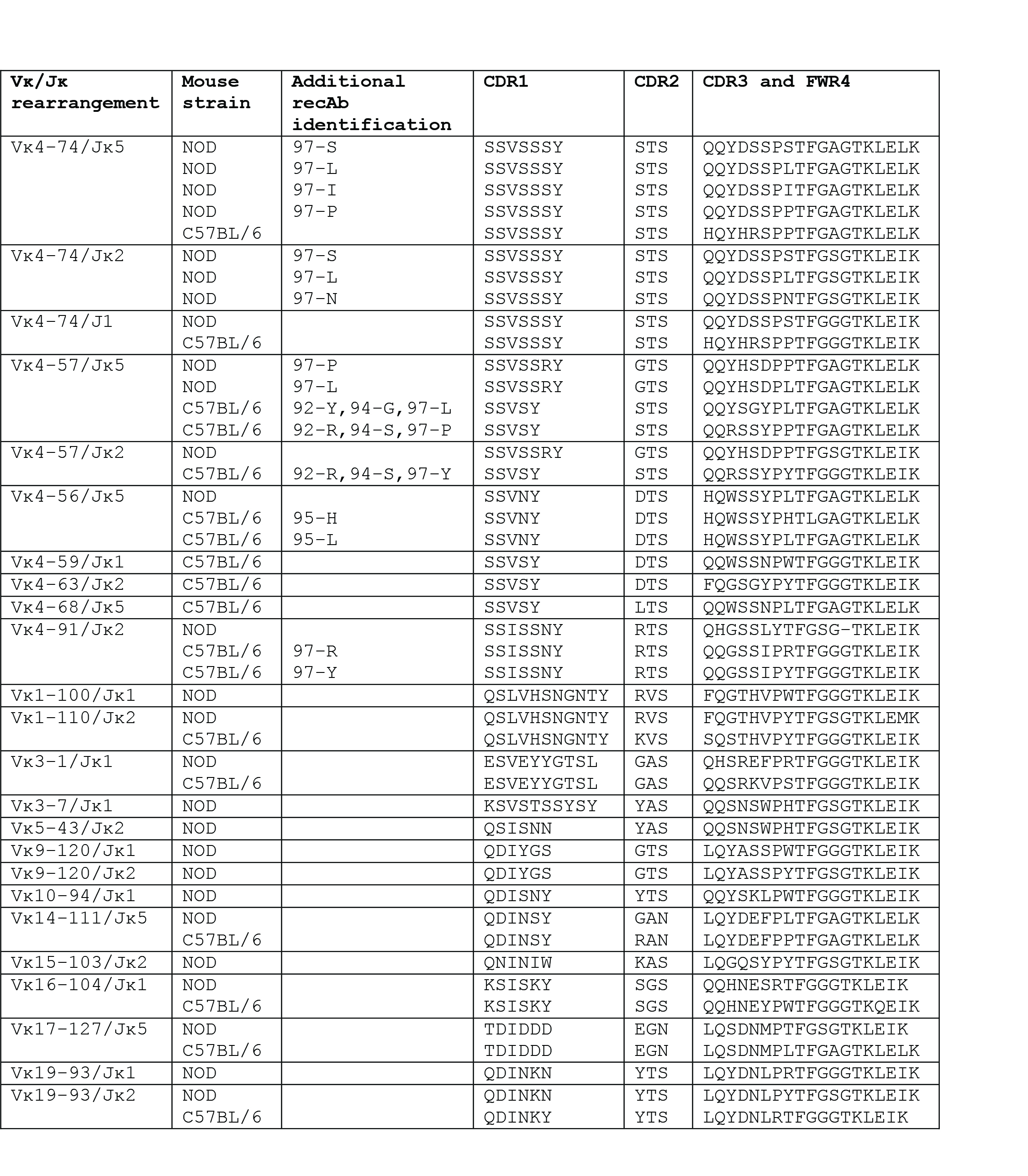

Supplement: Supplementary Table 2 — Amino acid composition of recombinant antibodies (recAbs) from VH125.NOD and VH125.C57BL/6-H2g7 genetic backgrounds. Amino acid composition of complementarity determining regions (CDR1-3) and framework 4 (FWR4) for recAbs composed of VH125 heavy chain and light chains derived from splenic IBCs of VH125.NOD (NOD) and VH125.C57BL/6-H2g7 (C57BL/6). Additional identification of amino acid differences in CDR3 is added for recAbs with the same Vκ/Jκ rearrangement. [file Image_5.jpg]
